# Metabolic responses and performance of Holstein × Gyr heifers grazing *Brachiaria decumbens* supplemented with varied crude protein levels

**DOI:** 10.1371/journal.pone.0289747

**Published:** 2023-08-24

**Authors:** Marcelo Messias Duarte Castro, Trevor James DeVries, Andreia Ferreira Machado, Marina Madureira Ferreira, Luciana Navajas Rennó, Marcos Inacio Marcondes

**Affiliations:** 1 Department of Animal Science, Universidade Federal de Viçosa, Viçosa, Minas Gerais, Brazil; 2 Department of Animal Biosciences, University of Guelph, Guelph, Ontario, Canada; 3 Department of Animal Sciences, Washington State University, Pullman, Washington, United States of America; Universidade Federal de Mato Grosso do Sul, BRAZIL

## Abstract

We aimed to evaluate the effect of supplemental CP on the nutritional characteristics and performance of Holstein × Gyr crossbreed heifers grazing intensively-managed *Brachiaria decumbens* throughout the year. Thirty-eight heifers with average initial body weight of 172.5 ± 11.15 kg (mean ± SE) and 8.2 ± 0.54 mo of age were randomly assigned to four treatments: three protein supplements (SUP) composed of soybean meal and ground corn fed at 5g/kg of BW, plus a control group (CON). The supplements had 12, 24 and 36% of CP for treatments **S12** (n = 9), **S24** (n = 10), and **S36** (n = 9), respectively. The experiment latest one year, subdivided into four seasons: rainy, dry, rainy-dry transition (RDT), and dry-rainy transition (DRT). Feces and pasture samples were collected for 4 days in each season, using chromium oxide, titanium dioxide, and indigestible neutral detergent fiber (NDF) to estimate fecal excretion, supplement, and pasture intake, respectively. The data were analyzed using PROC GLIMMIXED of the SAS with repeated measures. No effects of supplementation were detected on pasture and NDF intake. However, SUP animals had a greater intake of DM, metabolizable energy, and metabolizable protein. A positive linear response on metabolizable protein intake was observed among SUP animals. We observed an interaction between treatment and season for all digestibility variables, with a positive linear response in CP digestibility among SUP animals during all seasons. For neutral detergent fiber (NDF) digestibility, we observed a positive linear response in RDT and rainy seasons and a quadratic response during the dry season. Furthermore, SUP animals had greater average daily gain (ADG) than non-supplemented animals, and among SUP animals, there was a quadratic response to ADG, with the greatest gain observed in S24. We observed greater nitrogen retention coefficient in SUP animals than in non-supplemented animals and a positive linear effect among SUP animals. Supplemental CP did not affect microbial protein production and efficiency. We observed an interaction between treatment and season for blood glucose, with SUP animals having greater glucose concentration in all seasons than non-supplemented animals. Additionally, we observed a quadratic response among SUP animals only during RDT and dry season, with the greatest glucose concentration in S24. SUP animals had greater blood concentrations of urea and IGF-1. In conclusion, SUP animals had greater intake, digestibility, and performance than non-supplemented animals, with the 24% CP supplement demonstrating the best metabolic responses and performance.

## Introduction

The primary objective of a milk production system should be to maximize output while minimizing costs, ensuring high profitability without compromising cow health or welfare. Dairy farmers face significant expenses in rearing replacement heifers, including land, labor, and feed costs, with feed accounting for 64% of the total expenditure [1, 2]. To address this challenge, a pasture-based system emerges as a promising alternative that can reduce production costs and enhance profit rates [3, 4]. However, formulating balanced diets for grazing animals presents difficulties, as nutritional limitations often restrict intake, digestibility, and overall animal performance [5]. To overcome these limitations and enhance animal productivity, previous studies have explored the supplementation of crude protein (**CP**) for grazing cattle [6, 7].

Designing the best supplementation strategy and pasture management for grazing dairy heifers in tropical conditions is not a simple task since the pasture may have high variability in production and composition throughout the year. For example, pasture has low DM production during the dry season and is of lower nutritional quality, mainly due to low CP level and low fiber digestibility in tropical conditions [6, 8]. During seasons when pasture has low quality, the CP represents the first limiting for synthesizing enzymes involved in the fiber degradation process [6]. On the other hand, pasture has high dry matter (DM) production and better nutritive value during the rainy season compared to the dry season. However, there may still be an imbalance of nutrients in the pasture, mainly with surplus energy in relation to protein [7]. Thus, supplementation with adequate CP content is needed.

To ensure optimal animal performance and reduce the age at first calving, it is crucial to provide different CP supplementation strategies for grazing dairy heifers in each season. This is necessary due to the significant variability in production and pasture composition throughout the year [6]. However, existing studies on grazing animals have primarily focused on beef cattle, examining only one season, either dry or rainy [5, 7]. Consequently, there is a lack of research specifically addressing supplementation strategies for Holstein × Gyr crossbreed dairy heifers during grazing. It’s important to note that variations in animal nutrient requirements can be attributed to various factors, including environmental conditions, feeding management, and breed differences, which significantly contribute to response variability [9]. Therefore, supplementation strategies developed for beef cattle may not be suitable for achieving optimal performance and reaching the desired age at first calving for Holstein × Gyr crossbreed dairy heifers during grazing, considering their unique nutrient requirements. Therefore, it is of utmost importance to evaluate the responses of grazing Holstein × Gyr dairy heifers to supplementation throughout the year, encompassing the different seasons (rainy, rainy-dry transition, dry, and dry-rainy transition), in order to determine the most effective supplementation strategies for each season.

We hypothesize that there is an interaction between CP level in the supplement and season of the year for Holstein × Gyr grazing dairy heifers grazing intensively-managed *Brachiaria decumbens*, where high CP levels in the supplement composed of soybean meal and corn ground would promote a greater performance during the dry season. Therefore, the objective was to evaluate the effect of increasing CP supplementation on the metabolic characteristics and performance of Holstein × Gyr crossbreed heifers grazing intensively-managed *Brachiaria decumbens* throughout the year.

## Materials and methods

**Fig 1:**
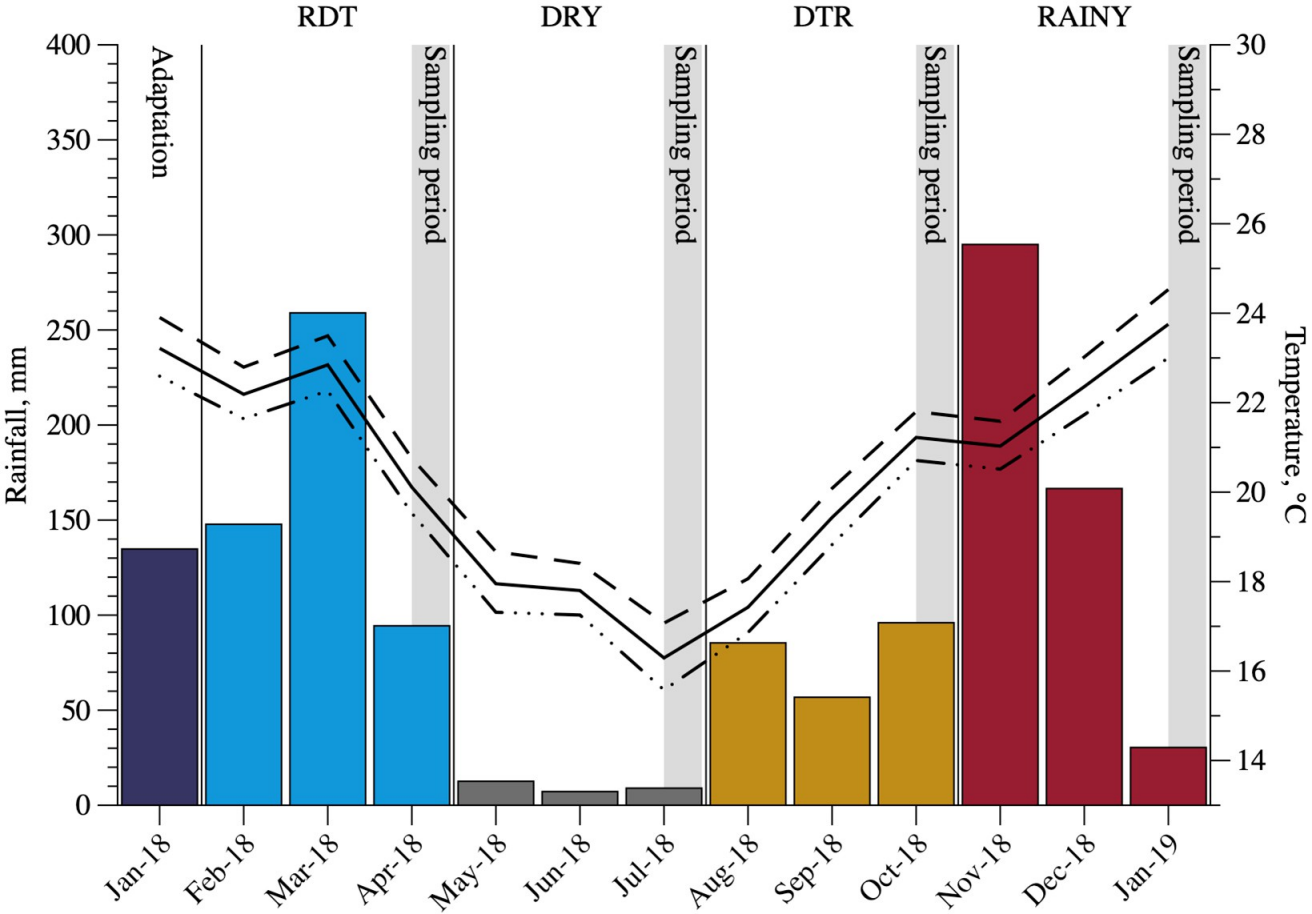
Rainfall (mm), minimum, average and maximum (°C) temperatures throughout seasons, rainy dry transition (RDT), dry, dry rainy transition (DRT) and rainy. Source: Department of Agricultural Engineering–Universidade Federal de Viçosa.

The experiment was carried out at the Department of Animal Science of the Universidade Federal de Viçosa (Viçosa, Minas Gerais, Brazil; 20º45’ S and 42º52’). Data for temperature and rainfall throughout the experimental period are presented in Fig 1. All animal handling and procedures were approved by the ethics committee for animal use at Universidade Federal de Viçosa under protocol #041/2017. The number of animals for this study was limited to that allowed by the ethics committee for animal use at Federal University Viçosa. The sample size was sufficient to detect a 20% difference (from control treatment) in the outcome variables (15% coefficient of variation), with 95% confidence at 90% power (WinPepi version 11.65; [10]).

### Treatments and experimental design

Thirty-eight crossbred heifers (½ Holstein × ½ Gyr), with average initial BW of 172.5 ± 11.15 kg (mean ± SE) and 8.2 ± 0.54 mo of age, were used. Initially, all heifers were treated for ectoparasites and endoparasites (Ivomec, Paulina, Sao Paulo, Brazil). The heifers were randomly assigned to 1 of 4 treatments: three supplements (SUP) had increasing levels of CP composed of soybean meal and corn ground (Table 1), plus a control group (**CON**; n = 10) in which the animals received only mineral mixture *ad libitum*. The supplements had 12, 24 and 36% of CP for treatments **S12** (n = 9), **S24** (n = 10) and **S36** (n = 9), respectively (Table 2). Every day, at 1000 h, all heifers were moved from the pasture to a management area (250 meters distance), and supplements were fed to each group (treatment) separately. However, we estimated the individual supplement intake using TiO_2_ as the external marker, following [11] methodology. Thus, the experimental unit was the animal. The supplement was supplied at 5 g/kg of BW per day with a feeder bunk space of 50 cm per animal, and no orts were observed. The amount of supplement (5g/kg of BW) was chosen to meet the energy requirements of crossbreed dairy heifers, with an ADG of 0.5 kg/d grazing *Brachiaria decumbens* according to recommendations of [12]. All heifers were weighed every 15 d to adjust the supplement supply. The animals of the control treatment (non-supplemented) were also moved to the management area, but no supplementation was provided. We spent 1 h with this management daily, and the animals returned to paddocks afterward.

**Table 1:** Proportion of ingredients and chemical composition of supplement for crossbred Holstein × Gyr heifers supplemented with different crude protein levels throughout the year.

Item	Adaptation^1^	Supplement^2^		
		S12	S24	S36
Ingredients, g/ kg of diet DM				
Ground corn	751	909	593	277
Soybean meal	249	91	407	723
Chemical composition, g/kg of diet DM				
Dry matter	909	907	912	918
Crude Protein	174	116	232	348
Neutral detergent fiber	116	104	128	150
Indigestible neutral detergent fiber	14.0	14.6	13.4	12.2
Ether extract	53.3	56.4	50.2	43.9
Non-fiber carbohydrate	618	694	543	395
Organic Matter	962	971	953	936
Total digestible nutrients^3^	815	844	787	789

^1^Supplemented provided to all heifers across the adaptation period;

^2^S12 = supplement formulated to contain 12% crude protein; S24 = supplement formulated to contain 24% crude protein and S36 = supplement formulated to contain 36% crude protein;

^3^Total digestible nutrients were calculated using the equation from NRC (2001).

**Table 2:** Production characteristics and chemical composition of pasture *Brachiaria decumbens* throughout experimental periods.

Item	Season			
	RDT^1^	Dry^2^	DRT^1^	Rainy^1^
Accumulated pasture (kg DM/ha per cycle)	1785	1080	1778	3092
Accumulated pasture (kg DM/paddock per cycle)	300	180	320	550
Pasture allowance (kg DM/animal per day)	8.00	9.67	8.45	14.4
Body weight (kg)	202	233	283	326
Forage pasture (g DM of herbage/kg BW)	39.6	41.4	29.8	44.0
Chemical composition (g/kg)				
Dry matter	173	283	255	226
Crude protein	108	77.8	86.8	100
Neutral detergent fiber	598	619	624	664
Undigestible neutral detergent fiber	143	200	191	173
Ether extract	40.2	32.7	37.2	38.5
Organic matter	900	920	924	923
Non-fiber carbohydrates	153	190	175	120

^1^during RDT, DRT and rainy season we used one paddock/day.

^2^during the dry season we used two paddocks/day.

RDT = rainy dry transition- February 7 to April 24.

Dry—April 25 to July 27.

DRT = dry rainy transition—July 28 to November 5.

Rainy—November 6 to January 22.

The heifers were managed in a rotational grazing system, with 27 paddocks, each 1700 m^2^, of *Brachiaria decumbens* fertilized with 120 kg of N and 60 kg of K_2_O per hectare per year. An additional area on the side of the main area was used during the dry season, with 23 paddocks, 1700 m^2^ each. All paddocks had free access to the resting area with shade (3 m^2^/animal), water, and mineral mix *ad libitum*. All heifers were kept in the same paddock, using one paddock per day, except during the dry season, when we used two paddocks per day (using the additional area described above) due to low pasture DM production in that season.

The experiment lasted from January 7 (2018) to January 22 (2019), subdivided into four seasons, plus an adaptation period of 30 days. The adaptation period lasted from January 7 to February 6, and all heifers were fed the same supplement containing 18% CP (Table 1) at 5 g/kg of BW. The first season lasted from February 7 to April 24 (76 d), which was named the rainy-dry transition (RDT) season. The second season lasted from April 25 to July 27 (94 d), which was named the dry season. The third season lasted from July 28 to November 5 (101 d) and was named the dry-rainy transition (DRT) season. Finally, the fourth season lasted from November 6 to January 22 (78 d), which was named the rainy season.

The last 14 d of each season were used to collect samples (collection period) of pasture, feces, urine, body measures, body weight, and blood samples (Fig 1). D 1 to 8 of the collection period were used to estimate digestibility, pasture intake, and supplement intake. From d 9 to 11, body measurements and body weight were recorded. Animals were weighed for three consecutive days (always at 0800 h) each period to avoid any scale or filling effects. On d 14, blood samples were collected.

### Herbage measurements, production, and composition

The pasture was managed under intermittent stocking. Pre-grazing herbage accumulation was estimated in each paddock on d 5 to 8 of each collection period, following the methodology described by [13]. Briefly, two 1.0 × 1.5 m (width × length) exclusion cages were placed in representative areas (based on height and morphological structure) immediately before beginning a new grazing cycle in each paddock. After that, pasture samples were collected at the actual post-grazing canopy height by clipping the area within the exclusion cages.

The pasture production was 1785, 1080, 1778, and 3092 kg of DM/ha, with average CP values (g/kg) of 108, 77.8, 86.8, and 100 for the RDT, dry, DRT, and rainy seasons, respectively (Table 2). Moreover, pasture allowance was 8.00, 9.67, 8.45, and 14.4 kg of DM/animal per day for the RDT, dry, DRT, and rainy seasons, respectively (Table 2).

### Feces and urine sampling

Between days 1 and 8 of each collection period, 10 g of titanium dioxide (TiO_2_) per kg of DM of supplement was mixed in the supplement to estimate the supplement intake of each animal [11]. On the same days, 15 g of chromium oxide (Cr_2_O_3_) was infused by the esophagic probe in each animal to estimate fecal excretion [14]. On d 5 to 8 of each experimental collection period, feces and urine samples were collected at 0600, 1000, 1200, and 1800 h, respectively. Approximately 300 g of feces were sampled directly from the rectum. The same procedures of drying and grinding done with forage samples were applied to fecal samples. In addition, urine samples were taken by vulva stimulation, and 50 mL of urine was sampled. To pool the urine, 10 mL of pure urine was diluted into 40 mL of sulfuric acid (0.036N) and stored at − 20ºC, to prevent purine derivatives degradation [15].

### Performance and body measurements

The heifers were weighed at the beginning of the experimental period and on days 9, 10, and 11 of each experimental collection period, always before supplementation, to calculate the ADG per period following [16] metodology. In addition, thoracic circumference, body length, rump length, rump width, withers height, and rump height were measured using a hipometer on the same days of the weighing in each experimental period following [16] methodology.

### Chemical analyses

Pasture and feces samples were oven-dried at 55º C for 72 h and then ground in a Wiley mill (TECNAL. Piracicaba. São Paulo. Brazil) with 2 mm and 1 mm using a knife mill [17]. Samples of feces, pasture, corn, and soybean meal ground at 1 mm were analyzed for DM [18] (method 930.15), OM [18] (method 924.05), CP [18], (method 984.13), EE [18] (method 920.39) and NDF [19] (method G-002/1). The samples ground at 2 mm were analyzed for undigestible NDF (uNDF). They were also incubated into the rumen of a ruminally-fistulated cow for 288 h, using non-woven textile bags (100 g/m^2^), and NDF was analyzed from the post-incubation material [20].

The non-fiber carbohydrates (**NFC**, g/kg) contents in the pasture, supplement, and feces were calculated as proposed by NRC [12].


NFC=OM-(NDF+CP+EE)

NFC = non-fiber carbohydrate (g/kg); NDF = neutral detergent fiber (g/kg) CP = crude protein (g/kg); OM = organic matter (g/kg) and EE = ether extract (g/kg).

Additionally, fecal samples were analyzed for chromic oxide (method INCT-CA M-005/1) and titanium dioxide (method INCT-CA M-007/1) according to [19].

Fecal DM excretion was estimated based on the ratio between the amount of the indicator provided (Cr_2_O_3_) and its concentration in feces [14]. Next, individual supplement intake was estimated using TiO_2_ as the external marker [11], using the equation:

$$DMSI=\frac{\left(FE\;\times MCF\right)}{MCS}$$

DMSI = dry matter supplement intake (kg/day); FE = fecal excretion (kg/day); MCF = marker concentration in the animal feces (kg/kg), MCS = marker concentration in the supplement (kg/kg).

Individual pasture intake was estimated using uNDF as the internal marker [14], using the equation:

$$DMPI=\frac{\left(FE\times CMF\right)-(CMS\times DMSI)}{CIF}$$

DMPI = dry matter pasture intake (kg/day); CMF = concentration of marker (uNDF) in feces (kg/kg); CIF = concentration of indicator(uNDF) in the forage (kg/kg); DMSI = dry matter supplement intake (kg/day); FE = fecal excretion (kg/day) and CMS = concentration of the marker (uNDF) in the supplement (kg/kg).

The urine samples were analyzed for N [18] (method 990.13) and creatinine was measured using the colorimetric endpoint method using picrate and acidifier (Labtest Diagnostica S. A. Lagoa Santa, Minas Gerais, Brazil). In addition, uric acid and allantoin concentrations in the urine were determined according to [21] and [22], respectively. Total daily urinary excretion was estimated using the daily creatinine excretion, as proposed by [23] for Holstein cattle. Ruminal microbial CP synthesis was estimated as a function of absorbed purines, which was calculated from the excretion of the purine derivatives uric acid and allantoin, according to the equations proposed by [24].

Metabolizable protein (MP) intake was calculated as true digestible microbial protein synthesis plus digestible rumen undegraded protein. The true fraction and digestibility of microbial protein were considered 80% [12]. Rumen-undegradable protein intake was estimated as the difference between CP intake and rumen-degradable protein intake, and its intestinal digestibility adopted for rumen-undegradable protein (RUP) was 80% [12].

### Blood sample and analysis

Blood samples were collected on the 13^th^ day of each collection period by puncturing the jugular vein. We used 10 mL vacutainer tubes containing separator gel and clot activator (silica) for serum collection and 5 mL tubes containing sodium fluoride for plasma collection. The tubes were kept on ice until centrifugation (3,000 × *g* at 4ºC for 20 min). Then, the serum and plasma were separately pipetted into Eppendorf tubes and stored (-20ºC) until analysis.

The serum sample was used to analyze blood urea nitrogen (BUN), total protein, albumin, total cholesterol, triglycerides, insulin and IGF-1, and the plasma sample was used for glucose analysis. Concentrations of BUN, glucose, total protein, albumin, total cholesterol, and triglycerides were measured by biochemical multi-analyzer (HumanStar 300; Human GmbH, Wiesbaden, DEU) with Bioclin kits. In addition, insulin and IGF-I were analyzed using a chemiluminescence immunoassay (Immulite 1000; Siemens Medical Solutions Diagnostics, Los Angeles, USA).

### Statistical analysis

The response variables were analyzed using the PROC GLIMMIX procedure of SAS (Statistical Analysis System University Edition). For all performance variables (BW and body measurements), measurements at d 0 were tested as covariates separately for each outcome variable and subsequently removed from the models as they were all non-significant (P > 0.05). The response variables were analyzed as a completely randomized design, and the season was included as a repeated measure in the model as follows:

$$Y_{ijk}=\mu \;+\;T_{i}+{\;\delta }_{ij}\;+\;S_{k}+{\left(T\times S\right)}_{ik}\;+\;\varepsilon_{ijk}$$

Where: Y_ijk_ = observation ijk; μ = the overall mean; T_i_ = fixed effect of treatment i; *δ*_ij_ = random error with mean 0 and variance $\sigma_{\delta }^{2}$, the variance between animals within the treatment and it is equal to the covariance between repeated measurements within animals; S_*k*_ = fixed effect of season k; T×P_ik_ = fixed effect of interaction between the treatment i and season k; and *ε*_ijk_ = random error with the mean 0 and variance *σ*^2^, the variance between measurements within animals. Fifteen variance-covariance structures were tested for each response variable. Then, we used the variance-covariance structure that provided the best fit based on the lowest Akaike information criterion. The variance components was the variance-covariance structure used. The observations with externally studentized residuals greater than |2.5| were first checked to make sure they were not a recording error. After checking and evaluating that the information was not a recording error, the observations were considered outliers and consequently excluded from the dataset. The analysis of possible outliers was performed only once for each outcome variable in order not to create erroneous results. After the outlier analysis, one animal was removed from the database as an outlier. The least-square means were considered different when *P* ≤ 0.05, and the tendency was used when 0.05 < *P* < 0.100.

Least square means (treatment effect) were compared by the following orthogonal contrasts: 1) effect of supplementation (non-supplemented animals-CON vs. S12 + S24 +S36) supplemented animals-SUP); 2) linear effect of supplement CP level; and 3) quadratic effect of supplement CP level. Those same contrasts were evaluated within each season in case of significant interactions.

## Results

### Intake and digestibility

Supplementation did not affect (Table 3) the intakes of pasture (kg/d and g/kg of BW), NDF, and uNDF (kg/day; *P* > 0.050). Animals with SUP had greater intakes (Table 3) of DM (kg/d and g/kg BW; *P* < 0.015), CP, and OM (kg/d; *P* < 0.050), metabolizable energy (Mcal/d; P < 0.001), and metabolizable protein (g/d; *P* < 0.001). Among SUP animal, there was no effect (Table 3) of CP level (P > 0.050) on intakes of pasture. DM (kg/d or g/kg BW), NDF, uNDF, OM (kg/d), and metabolizable energy (Mcal/d). Among SUP animals, we observed a linear positive response (Table 3) for CP (kg/d–*P* <0.001), RUP (g/d; *P* = 0.005) and metabolizable protein intake (g/d; *P* < 0.001).

**Table 3:** Least squares means for intake and apparent digestibility of nutrients of crossbred Holstein × Gyr heifers non-supplemented (CON) or supplemented with different levels of crude protein throughout the year.

Item	Treatments^3^				SEM	*P*-value				
	CON	S12	S24	S36		S	T × S^4^	SUP^5^	L^6^	Q^7^
Intake										
Supplement (kg/d)	—	1.17	1.21	1.09	—	—	—	—	—	—
Pasture (kg/d)	5.89	5.79	6.07	5.27	0.245	0.001	0.449	0.532	0.162	0.082
Dry matter (kg/d)	5.89	6.96	7.29	6.36	0.262	0.001	0.237	0.002	0.128	0.062
Pasture (g/kg of BW)	23.4	21.9	21.9	21.6	0.912	0.001	0.807	0.133	0.877	0.863
Dry matter (g/kg of BW)	23.4	26.1	26.4	26.1	0.977	0.001	0.721	0.015	0.983	0.852
Crude protein (kg/d)	0.56	0.68	0.85	0.88	0.030	0.001	0.117	0.001	0.001	0.060
Neutral detergent fiber (kg/d)	3.71	3.64	3.85	3.37	0.150	0.001	0.324	0.619	0.228	0.072
Indigestible neutral detergent fiber (kg/d)	1.03	1.05	1.10	0.94	0.042	0.001	0.506	0.997	0.111	0.055
Organic matter (kg/d)	5.40	6.35	6.62	5.76	0.237	0.001	0.309	0.003	0.103	0.062
Metabolizable energy (Mcal/d)	9.96	12.2	13.0	12.5	0.565	0.001	0.332	0.001	0.722	0.353
Rumen degradable protein (g/d)	392	388	337	423	32.4	0.001	0.999	0.798	0.471	0.089
Rumen undegradable protein (g/d)	233	326	492	475	36.7	0.001	0.398	0.001	0.005	0.051
Metabolizable protein (g/d)	437	508	609	651	26.1	0.001	0.274	0.001	0.001	0.364
CP: DOM (g CP/kg DOM)^1^^,^^2^^,^^5^	208	202	243	271	3.47	0.001	0.010	0.001	0.001	0.135
Apparent digestibility										
Dry matter (g/kg)^2^	457	499	499	539	5.96	0.001	0.001	0.001	0.001	0.009
Neutral detergent fiber (g/kg) ^6^	582	552	564	584	5.38	0.001	0.001	0.010	0.001	0.507
Crude protein (g/kg) ^2^	410	403	502	628	10.9	0.001	0.001	0.001	0.001	0.357
Organic matter (g/kg) ^2^	502	533	540	574	5.47	0.001	0.005	0.001	0.001	0.049

^1^DOM = digestible organic matter (apparent DOM was estimated in the total tract);

^2^CP:DOM = ratio between CP and DOM contents;

^3^CON = Control; S12 = supplement with 12% of CP; S24 = supplement with 24% of CP; S36 = supplement with 36% of CP;

**Fig 2:**
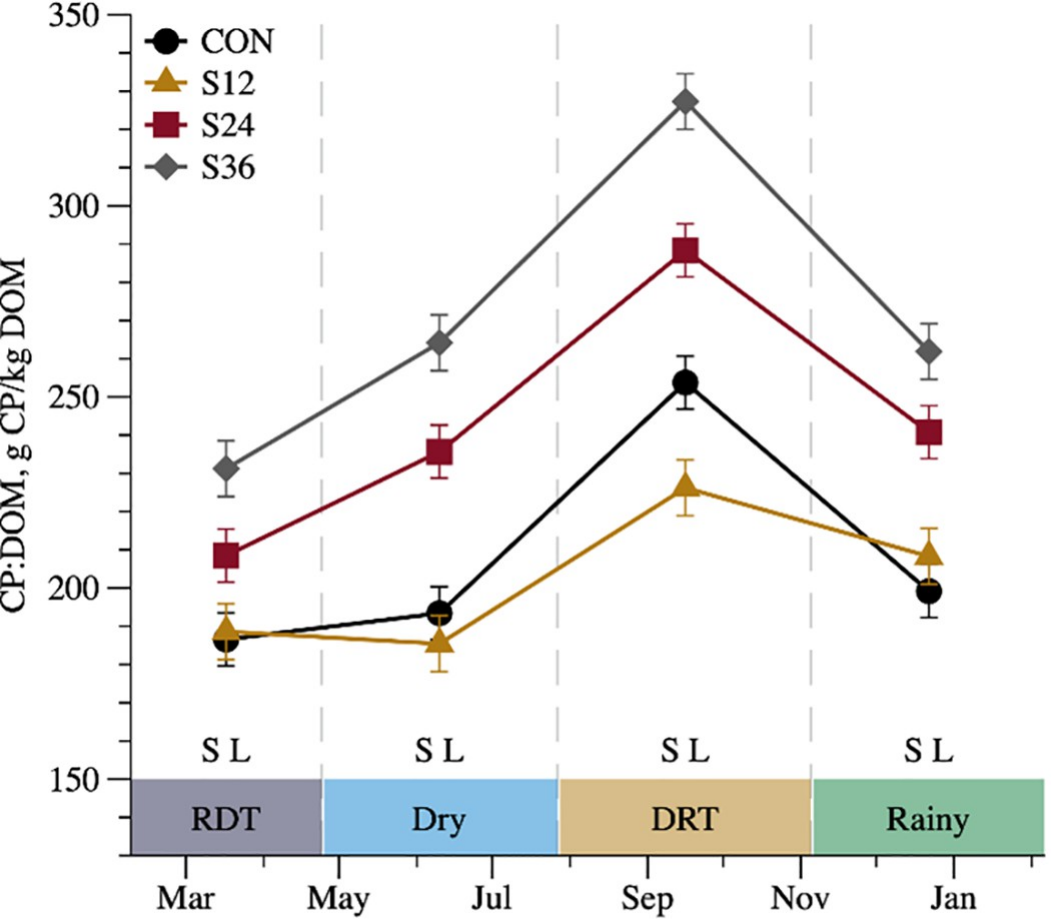
Interaction between treatment and season (P = 0.010) for ratio between crude protein and digestible organic matter (CP:DOM) in g CP/kg DOM of crossbred Holstein × Gyr heifers non-supplemented (CON) or supplemented with 12 (S12), 24 (S24) or 36% (S36) of CP in supplement throughout seasons, rainy dry transition (RDT—February 7 to April 24.), dry (April 25 to July 27), dry rainy transition (DRT—July 28 to November 5) and rainy (November 6 to January 22). S = supplemented vs non-supplemented effect (P < 0.050) and L = linear effect among supplemented animals (P < 0.050).

^4^S = season effect; T × S = interaction between season and treatment; SUP = supplemented *vs* non-supplemented; L = linear effect among supplemented animals; and Q = quadratic effect among supplemented animals;

^5^The interaction T × S for CP:DOM ratio is described in Fig 2;

**Fig 3:**
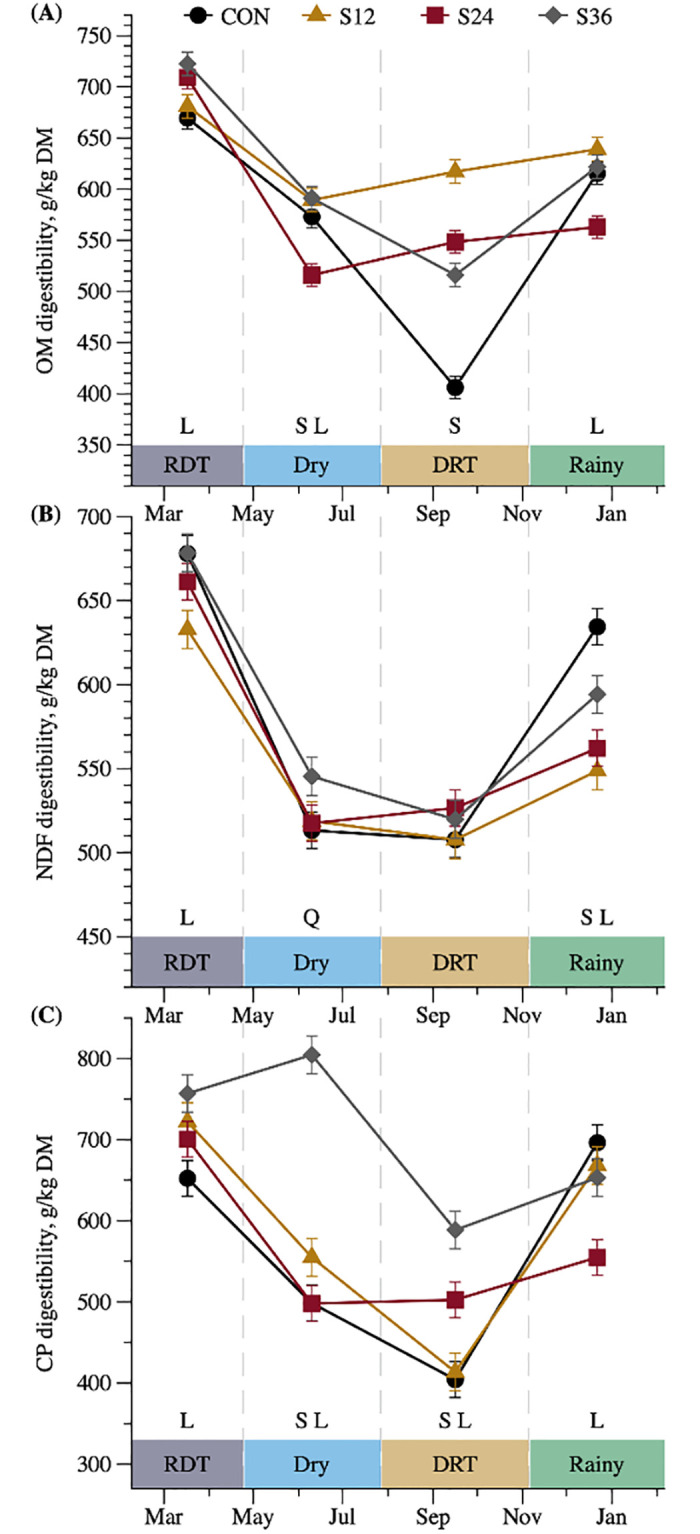
Interaction between treatment and season for apparent digestible coefficient of crossbred Holstein × Gyr heifers of organic matter(*P* = 0.005; OM- A), neutral detergent fiber (*P* < 0.001; NDF- B) and crude protein (*P* < 0.001; CP) throughout seasons, rainy dry transition (RDT—February 7 to April 24.), dry (April 25 to July 27), dry rainy transition (DRT—July 28 to November 5) and rainy (November 6 to January 22) in function of control (CON), supplement with 12% of CP (S12), supplement with 24% of CP (S24) and supplement with 36% of CP (S36). S = supplemented vs non-supplemented (*P* < 0.050), L = linear effect among supplemented animals (*P* < 0.050) and Q = quadratic effect among supplemented animals (*P* < 0.050).

^6^The interaction T × S for apparent digestibility of DM, neutral detergent fiber, CP and organic matter are described in Fig 3.

Moreover, for the relationship between CP and digestible OM (CP:DOM) intake, there was an interaction between treatment and season (*P* = 0.010; Table 3), in which the SUP animals had greater CP:DOM intake than non-supplemented animals in all seasons (Fig 2), but among SUP animals, there was a positive linear response of CP level in all seasons (Fig 2).

The results indicated an interaction between treatment and season (*P* < 0.05) for all digestibility coefficients (CP, NDF, and OM; Table 3). Supplemented animals had greater apparent digestibility of OM and CP in dry and DRT seasons (*P* < 0.05; Fig 3). However, non-supplemented animals had greater NDF digestibility in the rainy season (Fig 3). There was a positive linear response of CP level in CP digestibility among SUP animals during all periods (*P* < 0.050; Fig 3). There was a positive linear response to supplemental CP for OM digestibility in the RDT, dry, and rainy seasons (*P* < 0.05; Fig 3). For NDF digestibility, there was a positive linear response of CP supplement in the RDT and rainy seasons, and a convex quadratic response during the dry season (*P*< 0.05; Fig 3).

### Performance and body measurements

The SUP animals had greater final BW, withers height, thoracic perimeter, body length, and rump height than non-supplemented animals (*P* < 0.05; Table 4). However, there was no detected difference in final BW and body measurements among SUP animals.

**Table 4:** Final body measurements and final body weight of crossbred Holstein × Gyr heifers non-supplemented or supplemented with different levels of crude protein throughout the year.

Item	Treatments^1^				SEM	*P*–value^2^		
	CON	S12	S24	S36		SUP	L	Q
Final BW (kg)	307	338	345	316	0.828	0.008	0.060	0.068
Final withers height (cm)	118	121	123	121	0.893	0.001	0.923	0.071
Final thoracic perimeter (cm)	157	164	165	161	1.41	0.001	0.168	0.206
Final body length (cm)	131	134	138	137	1.49	0.006	0.461	0.246
Final rump width (cm)	43.8	44.7	46.1	44.0	0.924	0.293	0.611	0.143
Final rump length (cm)	45.2	46.8	46.5	45.6	0.754	0.199	0.262	0.776
Final rump height (cm)	123	125	127	125	0.926	0.044	0.719	0.084

^1^CON = Control; S12 = supplement with 12% of CP; S24 = supplement with 24% of CP; S36 = supplement with 36% of CP;

^2^SUP = supplemented *vs* non-supplemented; L = linear effect among supplemented animals; Q = quadratic effect among supplemented animals.

When evaluating relative performance, SUP animals had greater ADG, withers height gain, and thoracic perimeter than non-supplemented animals (*P* < 0.050; Table 5). In addition, among SUP animals, there was a quadratic response (*P* = 0.040) to ADG (Table 6), which was the greatest ADG observed in treatment S24 (Table 5).

**Table 5:** Average daily gain (ADG) and body measurements gain (mm/day) of crossbred Holstein × Gyr heifres non-supplemented (CON) or supplemented with different levels of crude protein throughout the year.

Item	Treatments^2^				SEM	*P*-value^3^				
	CON	S12	S24	S36		S	T × S	SUP	L	Q
ADG (kg/d)^1^	0.39	0.46	0.49	0.42	0.020	0.001	0.713	0.006	0.192	0.040
Withers height gain	0.41	0.51	0.57	0.51	0.033	0.001	0.567	0.003	0.890	0.178
Thoracic perimeter gain	0.73	0.92	0.95	0.85	0.044	0.266	0.671	0.001	0.284	0.251
Body length gain	0.49	0.60	0.70	0.66	0.074	0.001	0.115	0.063	0.602	0.439
Rump length gain	0.27	0.32	0.30	0.27	0.027	0.001	0.182	0.474	0.276	0.833
Rump width gain	0.28	0.31	0.35	0.30	0.026	0.002	0.282	0.157	0.555	0.136
Rump height gain	0.39	0.41	0.49	0.42	0.029	0.001	0.217	0.225	0.820	0.025

^1^ADG = average daily gain;

^2^CON = Control; S12 = supplement with 12% of CP; S24 = supplement with 24% of CP; S36 = supplement with 36% of CP;

^3^S = season effect; T × S = interaction between season and treatment; SUP = supplemented vs non-supplemented; L = linear effect among supplemented animals; and Q = quadratic effect among supplemented animals.

**Table 6:** Urine and nitrogen balance of crossbred Holstein × Gyr non-supplemented (CON) heifers or supplemented with different levels of crude protein throughout the year.

Item	Treatments^1^				SEM	*P*-value^2^				
	CON	S12	S24	S36		S	T × S	SUP	L	Q
Urea (mg/d)	236	237	375	583	19.5	0.001	0.696	0.001	0.001	0.130
Microbial protein (g/d)^3^	392	388	337	423	32.4	0.003	0.999	0.798	0.472	0.089
Microbial efficiency (g/kg DOM)^4^	177	124	104	146	14.0	0.001	0.314	0.002	0.292	0.082
Microbial efficiency (g/ kg CPI)	849	635	433	542	61.0	0.001	0.108	0.001	0.309	0.057
Nitrogen intake (g/d)	90.8	108	136	140	4.80	0.001	0.136	0.001	0.001	0.060
Nitrogen retained (g/d)	11.8	13.8	28.5	38.2	3.57	0.001	0.240	0.001	0.001	0.551
Retention coefficient (%)	4.57	8.64	18.5	23.0	2.17	0.001	0.062	0.001	0.001	0.329

^1^CON = Control; S12 = supplement with 12% of CP; S24 = supplement with 24% of CP; S36 = supplement with 36% of CP;

^2^S = season effect; T × S = interaction between season and treatment; SUP = supplemented vs non-supplemented; L = linear effect among supplemented animals; and Q = quadratic effect among supplemented animals;

^3^Microbial protein was estimated as a function of absorbed purines, which was calculated from the excretion of the purine derivatives (uric acid and allantoin);

^4^Apparent DOM was estimated in total tract.

### Urine and nitrogen balance

The results pointed a greater urea excretion (mg/d), nitrogen intake (g/d), nitrogen retention (g/d) and retention coefficient (g/kg) in SUP animals when compared with non-supplemented animals (*P* < 0.05; Table 6). In addition, non-supplemented animals had greater microbial efficiency in g/kg of digestible OM and g/ kg of CPI (*P* < 0.050; Table 6) than SUP animals.

Among SUP animals, there was a linear positive effect (*P* < 0.001) of CP supplementation on urea excretion, nitrogen intake, nitrogen retention, and retention coefficient (Table 6). However, the supplemental CP did not affect (*P* > 0.050) microbial protein production (g/d), microbial efficiency (g/kg of OM digestible), and g/ kg of CPI (Table 6).

Despite the observed results, microbial protein synthesis results should be carefully evaluated. Although functional, estimating microbial protein synthesis using purine derivates has intrinsic limitations. According to [25], the principal concern with purines is related to unequal purine-to-total N ratios in protozoal and bacterial pools coupled with the need to assume that dietary purines are completely degraded in the rumen [26, 27]. Furthermore, recent studies have shown that dietary purines can contribute between 13 and 33% of the purine flow in the duodenum of cattle, which would overestimate microbial protein synthesis [28, 29]. Thus, [25] mentioned that overall, calculating absolute changes in MPS based on the urinary purine derivatives is not advisable; however, for a controlled experimental setting (which is the case of this study), differences in excretion of total purine derivatives in urine could indicate differences in microbial protein synthesis. Thus, although the absolute microbial protein synthesis value observed in this study should not be used as a reference, considering that purine derivates are the most practical method to determine microbial protein synthesis in non-cannulated grazing animals, the variations among treatments could still render objective interpretations.

### Blood

**Fig 4:**
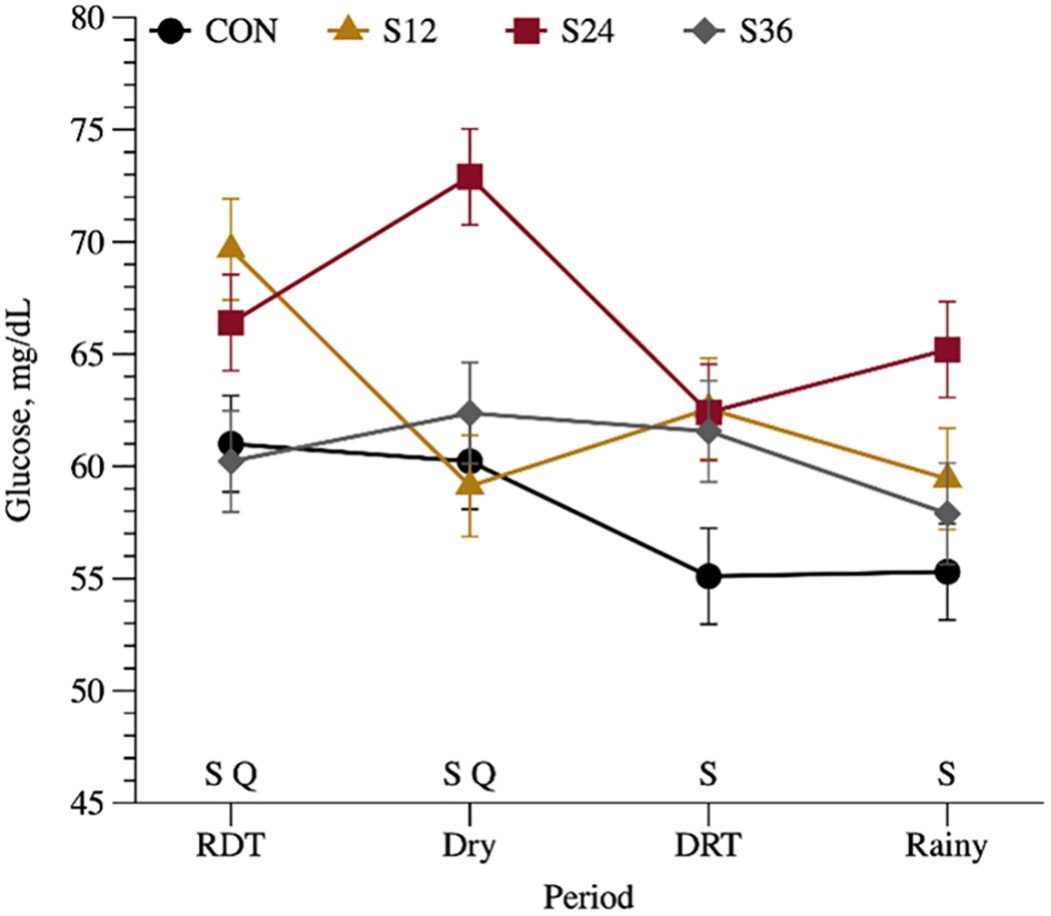
Interaction between treatment and season (*P* = 0.021) for glucose concentration (mg/dL) of crossbred Holstein × Gyr heifers non-supplemented (CON) or supplemented with 12 (S12), 24 (S24) or 36% (S36) of crude protein in supplement throughout seasons, rainy dry transition (RDT—February 7 to April 24.), dry (April 25 to July 27), dry rainy transition (DRT—July 28 to November 5) and rainy (November 6 to January 22). S = supplemented vs non-supplemented (*P* < 0.050) and Q = quadratic effect among supplemented animals (*P* < 0.050).

The results indicated an interaction between treatment and season (*P* = 0.021) for blood glucose (Table 7), where SUP animals had greater glucose concentration (*P* < 0.05) in all seasons than non-supplemented animals (Fig 4). Additionally, there was a quadratic response among SUP animals only during the RDT and dry seasons, with the greatest glucose concentration observed for S24 (Fig 4). No differences (P > 0.05). among SUP animals were detected in the other seasons.

**Table 7:** Blood parameters of crossbred Holstein × Gyr heifers non-supplemented (CON) or supplemented with different levels of crude protein throughout the year.

Item	Treatments^1^				SEM	*P*–value^2^				
	CON	S12	S24	S36		S	T × S	SUP	L	Q
Glucose (mg/dL)^1^	57.9	62.7	66.7	60.5	1.14	0.001	0.021	0.001	0.179	0.001
Cholesterol (mg/dL)	96.5	95.8	93.2	88.1	2.62	0.015	0.906	0.184	0.054	0.702
Triglycerides (mg/dL)	28.4	30.6	29.8	25.9	1.10	0.001	0.832	0.763	0.003	0.233
Total Protein (mg/dL)	7.20	7.44	7.43	7.45	0.121	0.001	0.132	0.087	0.893	0.954
Albumin(mg/dL)	2.98	3.02	2.97	3.02	0.056	0.001	0.545	0.729	0.969	0.455
Globulin (mg/dL)	4.22	4.44	4.46	4.43	0.112	0.005	0.222	0.078	0.096	0.840
Urea (mg/dL)	15.0	16.4	19.5	27.6	1.09	0.001	0.275	0.001	0.001	0.056
IGF-1(mg/dL)	163	183	208	201	9.33	0.001	0.948	0.001	0.181	0.138
Insulin (ng/mL)	1.55	1.04	2.21	2.12	0.465	0.302	0.385	0.637	0.106	0.250

^1^CON = Control; S12 = supplement with 12% of CP; S24 = supplement with 24% of CP; S36 = supplement with 36% of CP;

^2^S = season effect; T × S = interaction between season and treatment; SUP = supplemented vs non-supplemented; L = linear effect among supplemented animals; and Q = quadratic effect among supplemented animals;

^3^The interaction T × S for glucose concentration is described in Fig 4.

Supplemented animals had greater blood concentrations (*P* > 0.05) of urea and IGF-1 (Table 7) than non-supplemented animals. Among SUP animals, there was a negative linear response to CP supplementation in triglycerides (*P* = 0.003) and a positive linear response (*P* < 0.001) to urea (Table 7). For cholesterol, triglycerides, total protein, albumin globulin, and insulin, there were no detected differences (*P* > 0.05) between SUP and non-supplemented animals (Table 7). In addition, we did not observe differences (*P* >0.050) in blood cholesterol, total protein, albumin, globulin, IGF-1, and insulin among SUP animals (Table 7).

## Discussion

### Pasture measurements

In this study, the high pasture availability (g/kg of BW) during the dry season was due to the utilization of two paddocks per day because of the low DM production per hectare during that season. [30] suggested offering at least 40 to 50 g/kg BW of potentially digestible DM (pdDM) to promote maximum performance of grazing animals (in studies with beef animals). The present study’s pasture availability was 39.58, 42.34, 29.79, and 44.04 g/kg of BW during the RDT, dry, DRT, and rainy seasons, respectively. The values observed in the present study are within the range suggested by [30], given that researchers estimated pasture availability by cutting sampling forage at the ground level, while we determined pasture availability by sampling the grazing stratum [13]. Thus, we assume that our animals had no limitation of pasture intake during all evaluated seasons.

### Intake and digestibility

Previous studies have described the benefits of CP supplementation to pasture-fed cattle on DMI since it improves the rumen environment by providing substrate (carbon, peptides, and amino acids) for the growth of fibrolytic bacteria, increasing fiber digestibility and, consequently, pasture intake [31, 32]. However, other studies have also highlighted that N supplementation mainly stimulates pasture intake when the forage CP level is less than 70 g/kg DM of pasture [33]. In our study, the lowest pasture CP level observed was 77.89 g/kg of DM (during the dry season, Table 8), thus explaining why there was no difference in pasture intake among the treatments (Table 3). In this sense, it can be concluded that the supplementation level used in this study (5 g/ kg of BW) had no beneficial effect on pasture intake, but also did not cause a substitutive effect on pasture intake.

**Table 8:** Crude protein (CP) level in diet (g/kg of dry matter) throughout the experiment period for crossbred Holstein × Gyr heifers non-supplemented (CON) or supplemented with different levels of crude protein throughout the year.

Season	Treatments^1^			
	CON	S12	S24	S36
^2^RDT	108	109	125	144
^3^Dry	78.0	84.5	106	128
^4^DRT	86.8	92.8	114	136
^5^Rainy	100	103	121	140

^1^CON = Control; S12 = supplement with 12% of CP; S24 = supplement with 24% of CP; S36 = supplement with 36% of CP;

^2^RDT = rainy dry transition- February 7 to April 24.

^3^Dry—April 25 to July 27.

^4^DRT = dry rainy transition—July 28 to November 5.

^5^Rainy—November 6 to January 22.

Despite no differences in pasture intake, the supplementation promoted greater DMI (kg/d and g/kg of BW). Consequently, non-supplemented animals had lower metabolizable energy (Mcal/d) and protein (g/d) intakes than SUP animals. Regardless of treatments, all our diets were high in NDF due to the high concentration of pasture NDF (average 629.3 g/kg), which likely affected the intake limit [34]. However, we highlight that there were no differences in NDF intake between SUP animals and non-supplemented animals and among SUP animals with increasing levels of CP (Table 3). Moreover, despite the large numerical difference (no statistical analyse) in the levels of EE and NFC among supplement (Table 1) due to ingredients used in its formulation (soybean meal and ground corn), when we simulated the diets in the NRC 2001 program, these differences were not enough to ensure a difference in energy intake among SUP animals, as demonstrated in Table 3.

The relationship between CP and DOM is an indicator of the protein:energy dietary ratio and might be linked to the metabolic effects of protein on intake. The synchronism between protein and energy may promote the maximum microbial production and reduce N losses via ammonia and energy of carbohydrates, promoting digestion improvements, mainly NDF digestibility [35]; hence, increasing the availability of metabolizable energy and protein for animal growth. Thus, the CP:DOM (gCP/kgDOM) works as a regulating parameter, directly influencing voluntary DMI [6, 36]. In the present study, the CP:DOM ratios observed were 208.2, 202.2, 243.3, and 271.1 g of CP/kg DOM for CON, S12, S24, and S36, respectively. Non-supplemented animals had a lower CP:DOM ratio than SUP animals in all seasons (Fig 2), with an average value (208.2 g/kg DOM) lower than that suggested for optimizing DMI which could explain once again the lower DMI in non-supplemented animals than SUP animals. Among SUP animals, we observed a trend of quadratic response for DMI, where the highest DMI was observed for S24. Dietary CP might explain this quadratic response in CP:DOM ratios in SUP animals, which showed a linear response in all seasons (Fig 2). The lowest DMI observed in S12 and S36 than S24 may be explained by different reasons. First, the low CP:DOM ratio in S12 was below the minimum suggested by [6] and [37]) for maximum DMI (210 g/kg). Second, the S36 treatment likely had excess CP relative to the energy available. This excess of N in relation to energy (CP:DOM) has consequences on animal metabolism, leading to greater heat production [36], deficiency of ATP in liver metabolism due to a high rate of the utilization urea cycle [37], and animal indisposition due to excess ammonia in the blood [6]. Indeed, our S36 animals had greater urea excretion, which indicates that some of those metabolic pathways were in play in those animals. Additionally, a quadratic response in intake due to increased CP supplementation was also observed in other studies [38, 39]. Corroborating with the results presented in this study, we simulated the experimental diets in a formulation program [12], and observed that S36 had greater metabolizable protein available for gain than metabolizable energy available for gain. Similar values were observed for S24. Lastly, greater metabolizable energy available for gain than metabolizable protein, was observed for S12.

The digestibility coefficient of nutrients can be influenced by factors such as pasture quality, level intake, chemical compositions of the feed, and others. CP supplementation improved overall nutrient digestibility in our study, mainly in the seasons where pasture showed the worst nutritional values (Fig 3). This is supported by the greater digestibility coefficient of the SUP animals for DM and OM in the dry, DRT, and rainy seasons, which had a poorer quality pasture (Table 2). Corroborating these results, SUP animals had greater CP digestibility in the dry and DRT seasons. Further, we observed a positive linear effect among the SUP animals in almost all seasons for DM, OM, NDF, and CP, except for DM, OM, and NDF in the DRT season and NDF in the dry season.

The benefits of supplementation were increased in seasons where forage had the lowest CP content (dry, DRT, and rainy), indicating the benefits of CP supplementation for grazing cattle. Previous studies have shown that the benefits of CP supplementation are increased when forage has low CP content [7, 40], mainly increasing nutrient intake and digestibility coefficients [41, 42]. In our case, the improvement in digestibility is caused by the increase in the supply of essential substrates (N and starch), minimizing the deficiencies of nutrients required by ruminal microorganisms. Nitrogen is likely the most important nutrient in this case since it is involved in the synthesis of enzymes linked to the degradation of fiber in low-quality forages [6, 31].

### Performance and body measurements

We observed that the animals SUP had greater ADG, withers height, and thoracic perimeter gain (mm/d) than non-supplemented animals. Consequently, SUP animals had greater final BW weight, final thoracic perimeter, final body length, final withers, and rump height. In general, SUP animals had greater nutrient intake and digestibility. Consequently, they had greater metabolizable energy and protein intake, which resulted in better performance [43].

We observed a quadratic effect for final body weight, final thoracic perimeter, and ADG in SUP animals, with S24 animals having the best performance. The lowest performance was observed in the S12 and S36 animals and might be explained by deficiency and excess of N in those treatments, respectively. Previous studies carried out with supplementation of cattle in pasture also have observed quadratic responses to N supplementation [38]. As mentioned before, in a tropical pasture system, the first limiting nutrient is N [6, 44], which might have limited microbial growth and subsequent intake, digestibility, and performance [44, 45] of the S12 animals. Hence, we observed the lowest dietary CP, MP intake, CP:DOM ratio, and digestibility in S12 animals compared to only SUP animals.

The decline in the performance of S36 animals may be related to the excess of dietary CP since there is a need to excrete it as urea via urine, and this process increases heat productionv [46]. Additionally, increased ammonia level in the liver due to excessive-high dietary CP is linked to decreased levels of NADPH, NADP, and NADH, due to competition for ATP between the urea cycle and other gluconeogenic pathways [12], thus damaging energetic metabolism in the body [47] and ATP synthesis in the liver and other tissues. Moreover, there was a decrease in DMI in the S36 animals in our study. High N diets usually involve increased heat production due to increased urea cycling. This could lead to extra energy used for dissipation of this additional heat increase and augmented heat stress, impacting animals’ performance [38].

### Urine and blood

The greater N intake in SUP animals promoted a greater blood urea concentration and, consequently, greater urea excretion (mg/d) than in non-supplemented animals. Moreover, SUP animals were more efficient than non-supplemented animals regarding N metabolism since they had greater retained nitrogen and retention coefficient. Once again, these results highlighted the beneficial effects of N supplementation, which improved N status in the body and N components balance between rumen and bloodstream, consequently improving animal performance [48]. Furthermore, we observed higher glucose and IGF-1 in SUP animals than in non-supplemented animals. Therefore, the benefits of N supplementation go beyond their effects on intake and digestibility, such as improved energy and nitrogen status, which resulted in greater levels of IGF-1 and glucose.

A positive linear response of supplemental CP was detected for N intake, retained N, and retention coefficient among SUP animals. Furthermore, Detmann et al. [6] observed a positive relationship between CP supplementation and N retention. Thereby, the supplementation with N allowed for greater availability of N for all metabolic and physiological processes in the body. Nitrogen supplementation causes a negative impact on the breakdown rate of myofibrillar protein and positively affects N and anabolic hormone concentrations in the blood (e.g., IGF-1) [49, 50]. These responses resulted in increased nitrogen retention in the animal’s body.

Interestingly, microbial protein production estimates (g/d) were not different between SUP and non-supplemented animals. However, SUP animals had lower microbial efficiency in g/kg of digestible OM and g/kg of CPI. Thus, we observed an increase in the efficiency of non-supplemented animals because of a severe deficiency in rumen N or ammonia. Thus, non-supplemented animals highly depended on recycled N to keep microbial activity in the rumen environment [6], increasing their efficiency per N intake unit. Furthermore, in this situation, the animals decrease their urinary N excretion and increase the recycling of dietary N within the rumen [51]. Lastly, animals may mobilize protein from tissue in N deficient diet to keep the amount of recycled N [12], with a consequent flow of N to the abomasum greater than N intake [6].

Curiously, we did not observe an effect of supplemental CP on microbial protein production in g/d, microbial efficiency in g/kg of digestible OM and g/kg of CPI among the SUP animals. Similar results were observed by [31] and [48], highlighting that increasing N content alone is not enough to ensure an increase in microbial protein synthesis. In our study, despite the similar energy level in the diets (TDN–Table 1) among SUP animals, the increase in the CP:DOM ratio (Table 3) was not enought to ensure an increase in microbial protein synthesis.

As mentioned earlier, the animals supplemented with SUP exhibited higher glucose blood concentration compared to the non-supplemented animals. This difference can primarily be attributed to the increased intake and digestibility of nutrients in the SUP group, leading to higher levels of glucogenic precursors in the bloodstream. Our concentrates were rich in corn, which stimulates propionate production in the rumen. Propionate is then transported to the liver and converted into glucose, thereby increasing the availability of gluconeogenic precursors [52]. Additionally, N supplementation improves the N status in the body, reducing the need for amino acid catabolism for urea synthesis in the liver [53]. By doing so, N supplementation decreases the utilization of gluconeogenic amino acids for urea synthesis, thereby enhancing their availability for glucose synthesis. Consequently, this contributes to the elevated glucose content in the body of SUP animals.

During the RDT and dry season, we observed a quadratic effect among SUP animals, with the highest glucose concentration in the S24 animals. This can be attributed to their increased intake of DM and OM, resulting in greater consumption of gluconeogenic precursors. IGF-1 is an endocrine regulator of muscle growth in cattle, playing a vital role in the metabolic growth process, particularly in skeletal muscle [54]. The concentration of IGF-1 is typically elevated in response to dietary changes, with a more pronounced response to dietary protein concentration rather than energy concentration [55]. Moreover, SUP animals demonstrated higher serum concentrations of IGF-1 compared to the non-supplemented animals, which aligns with similar findings reported by [32] and [56]. Interestingly, the increase in IGF-1 was also associated with elevated glucose concentration, potentially accelerating the anabolic stimulus for body protein synthesis. This may explain the higher nitrogen retention and retention coefficient observed in SUP animals, as mentioned earlier.

Among SUP animals, we observed a linear negative response for blood triglyceride concentration with increased supplement CP. Blood triglycerides in growing heifers usually originate from body reserves or diet mobilization. The SUP animals were in a positive energy balance in the present study. In this sense, the range in blood triglyceride concentrations observed can only be explained by diet composition variation or the proportion of each ingredient (ground corn and soybean meal) used in the supplement composition. The supplements with higher CP had a greater proportion of soybean meal. This switch likely caused a slight decrease in supplemental energy density, which may have caused the observed decrease in blood triglycerides.

## Conclusion

The supplementation of grazing dairy heifers improved DMI, OMI, ratio CP:OM, energy, and protein metabolizable intake. Moreover, supplementation increased the digestibility coefficient of DM and OM in the dry, DRT, and rainy seasons and CP in the dry and DRT seasons. In addition, the CP supplementation resulted in greater levels of glucose and IGF-1 and improved N balance. Consequently, SUP animals had greater performance than non-supplemented animals. Among SUP animals, we observed the best results in animals supplemented with 24% CP in the supplement, which had the greatest ADG, DM, and OM intake and glucose concentration in the RDT and dry seasons. Therefore, the supplementation with 5 g/kg of BW of supplement composed of soybean meal and ground corn for Holstein × Gyr crossbreed heifers grazing intensively managed *Brachiaria decumbens* throughout the year promoted greater levels of intake and digestibility, improved nitrogen balance, and animal performance, and 24% of CP showed the best results among supplemented animals. However, we emphasize that further research should be carried out with grazing animals throughout the seasons to validate the observed results.

## Supporting information

S1 Data (XLSX)
